# Brain Stem Glioma Recurrence: Exploring the Therapeutic Frontiers

**DOI:** 10.3390/jpm14090899

**Published:** 2024-08-25

**Authors:** Francesco Pasqualetti, Giuseppe Lombardi, Giovanni Gadducci, Noemi Giannini, Nicola Montemurro, Alberto Feletti, Marco Zeppieri, Teresa Somma, Maria Caffo, Chiara Bertolotti, Tamara Ius

**Affiliations:** 1 Division of Radiation Oncology, Azienda Ospedaliero Universitaria Pisana, 56100 Pisa, Italy; francep24@hotmail.com (F.P.);; 2 Department of Oncology, Oncology 1, Veneto Institute of Oncology IOV-IRCCS, 35128 Padua, Italy; 3 Department of Neurosurgery, Azienda Ospedaliero Universitaria Pisana, 56100 Pisa, Italy; 4 Department of Neurosciences, Biomedicine, and Movement Sciences, Institute of Neurosurgery, University of Verona, 37126 Verona, Italy; 5 Department of Ophthalmology, University Hospital of Udine, Piazzale S. Maria della Misericordia 15, 33100 Udine, Italy; 6 Division of Neurosurgery, Department of Neurosciences, Reproductive and Odontostomatological Sciences, Federico II University, 80134 Naples, Italy; 7 Unit of Neurosurgery, Department of Biomorphology and Dental Science, and Morphofunctional Imaging, Università degli Studi di Messina, 98125 Messina, Italy; 8 Department of Neuroradiology, University Hospital of Udine, p.le S. Maria della Misericordia 15, 33100 Udine, Italy; 9 Neurosurgery Unit, Head-Neck and NeuroScience Department, University Hospital of Udine, p.le S. Maria della Misericordia 15, 33100 Udine, Italy

**Keywords:** brain stem, glioma, tumor recurrence, survival, radiotherapy, retreatment

## Abstract

Gliomas of the brainstem represent a small percentage of central nervous system gliomas in adults. Due to the proximity of the tumor to critical structures, radical surgery is highly challenging and limited to selected cases. In addition, postoperative treatments, which become exclusive to non-operable patients, do not guarantee satisfactory disease control, making the progression of the disease inevitable. Currently, there is a lack of therapeutic options to control tumor growth after the diagnosis of recurrence. The rarity of these tumors, their distinct behavioral characteristics, and the limited availability of tumor tissue necessary for the development of prognostic and predictive biomarkers contribute to the absence of a standardized approach for treating recurrent brainstem gliomas. A salvage radiotherapy (RT) retreatment could represent a promising approach for recurrent brainstem gliomas. However, to date, it has been mainly evaluated in pediatric cases, with few experiences available to assess the most appropriate RT dose, safety, and clinical responses in adult patients. This comprehensive review aims to identify instances of adult patients with recurrent brainstem gliomas subjected to a secondary course of RT, with a specific focus on the analysis of treatment-related toxicity and outcomes. Through this investigation, we endeavor to contribute valuable insights into the viability and efficacy of salvage RT retreatment in managing recurrent brainstem gliomas in the adult population.

## 1. Introduction

Adult brainstem gliomas (BSGs) are a highly heterogeneous group of tumors. While they are modestly represented in children (constituting approximately 10% of all pediatric brain tumors), their occurrence in adults is relatively rare, accounting for only 1–2% of all brain tumors [1]. Among adults, brainstem tumors predominantly affect the pons, comprising approximately 60–63% of cases, although they can also be found in the medulla oblongata (25%) and the midbrain (12–15%) [2,3]. In around 80% of cases, a combination of these brainstem structures is affected [2,3]. Despite clinical presentation and radiographic appearance similarities, adult brainstem lesions encompass a diverse range of entities. Although glial tumors are the most common, other considerations include brainstem metastases, lymphomas, and infectious or inflammatory lesions. Distinguishing among these entities based solely on radiological characteristics can pose challenges [4,5]. Based on clinical and radiographic characteristics, we can subdivide brainstem gliomas into diffuse intrinsic low-grade brainstem gliomas, focal and malignant brainstem gliomas, focal and tectal gliomas, and exophytic growing tumors [2]. Notably, most of the existing studies on brainstem gliomas in adults have employed varying classification schemes, thereby complicating the comparison of findings across studies. Nonetheless, these studies indicate that survival and overall prognosis are influenced by factors such as underlying pathology, pathological grade, and other clinical variables. A recent and comprehensive study revealed statistically significant differences in overall survival (OS) based on pathological grade, imaging characteristics, and age at diagnosis. Specifically, tumors classified as WHO grade 4 and those exhibiting contrast enhancement on magnetic resonance imaging (MRI) were associated with a more unfavorable prognosis.

Interestingly, a midbrain location showed a trend toward improved survival in this investigation [3]. Furthermore, while brainstem gliomas in adults are considered malignant tumors, the overall prognosis appears to be more favorable compared to brainstem gliomas in children. In pediatric cases, up to 80% of brainstem tumors are highly aggressive diffuse intrinsic brainstem gliomas.

Unlike gliomas arising in other parts of the central nervous system, mutations in the IDH1 or IDH2 genes are not common in patients with BSG; the literature has only described a few cases to date (accessed on 1 January 2024). The latest 2021 WHO classification of CNS tumors has provided a more precise delineation of brainstem tumors by introducing pontine midline tumors associated with the H3K27 mutation [6,7]. This genetic alteration affects the enzymatic function of EZH2, a critical component of the Polycomb Repressive Complex2 (PRC2), which plays a fundamental role in DNA structure within nucleosomes [8]. In pediatric patients, the H3K27M mutation is particularly relevant and has been linked to heightened tumor aggressiveness and reduced responsiveness to standard therapies, including radiotherapy (RT) [9]. Its presence can significantly impact patient prognosis, often necessitating more aggressive or alternative treatment approaches. Recent research has revealed the coexistence of other mutations in these tumors, conferring resistance to current therapies. Wang et al. reported an analysis of 96 adult diffuse intrinsic pontine gliomas; the frequencies of H3K27M and IDH1 mutations were 37.2% and 26.5%, respectively. This underscores the heterogeneity of brainstem tumors and suggests the potential for further sub-classification based on molecular characteristics [10]. Such advancements promise to improve patient stratification, refine prognosis, and guide targeted therapeutic interventions.

Diagnosing and treating BSGs have greatly benefited from conventional brain MRI [11,12,13]. Specifically, the distinctive feature of diffuse infiltrative pontine gliomas justifies treatment, avoiding invasive methods to acquire histological validation [14]. The prognostic role of contrast enhancement uptake shown by MRI still needs to be fully understood. Some research linked the presence of contrast enhancement to survival [11]. Dellaretti et al. conducted a retrospective study on 100 patients with BSG, including 63 adults and 27 pediatric patients. Patients who had enhancing lesions on MRI after contrast injection had a median survival of 21.7 months, while those without enhancing lesions had a median survival of 54.2 months (*p* < 0.001) [4].

Furthermore, studies reported the results of a retrospective study evaluating patients diagnosed with intrinsic pontine glioma [13]. In their analysis, contrast-enhancing lesions on MRI were associated with patient survival (*p* = 0.002). However, other authors found that contrast enhancement was not necessarily linked to a worse ending. Ueoka et al. (2009) reported data from a retrospective study of 86 patients with BSG. The authors found no differences in the time to early or late recurrence (less than or greater than 12 months) based on the presence or absence of contrast enhancement on MRI [13]. Diffuse pontine gliomas often do not enhance but can nevertheless be very aggressive, in contrast to supratentorial gliomas, where contrast enhancement typically indicates a higher tumor grade and a shorter survival time [13]. The more prolonged survival in this population is probably due to lower-grade BSGs with a less prominent preferential location at the pons in adults.

Some mistakes that can occur in BSG diagnosis highlight the need to consider more systematic use of a biopsy in this setting whenever practically feasible, as is currently done in the pediatric population by skilled surgical teams, with relatively low morbidity and mortality [14,15,16]. Similarly, positron emission tomography (PET) imaging can be used in this kind of tumor. Abdullah et al. [17] showed that PET uptake of 18F-fluoroethyl-L-tyrosine may be correlated with disease progression in adult BSGs, as the more intense the fixation, the worse the prognosis.

The rarity of these tumors in adults poses challenges in identifying prognostic factors and predictors of treatment response. For example, in 2001, Guillamo et al. [2] conducted a translational study involving 48 adult patients with brainstem gliomas to investigate prognostic factors. The researchers examined various clinical variables and identified several positive prognostic indicators (*p* < 0.01). These included onset age below 40 years, symptom duration exceeding three months before diagnosis, Karnofsky performance status above 70, low-grade tumor histology, absence of contrast enhancement on imaging, and the presence of “necrosis” on MRI scans (Table 1). In the multivariate analysis, negative prognostic indicators reported included prolonged symptom duration, presence of “necrosis” on MRI scans, and a severe histological grade of the tumor (*p* < 0.05).

Following the radiological diagnosis of these malignancies, clinicians must evaluate the feasibility of a surgical approach for both cytoreductive and diagnostic purposes [18,19,20,21,22,23,24,25,26,27,28,29,30]. However, considering the critical structures surrounding the tumors, not all patients are suitable for surgery. Since performing radical resection is complex and these tumors tend to relapse, RT is an essential part of the treatment of these gliomas after surgery or as the exclusive treatment [20]. Based on retrospective studies and small case series, the standard treatment for adult BSG patients is a dose of 50–60 Gy with conventional fractionation [18]. This dose is well tolerated and controls the lesion without recurrence for a longer or shorter amount of time, depending on the histology of the lesion. Yu et al. [22] chose to treat their adult patient with BSGs with combined-modality management despite the existence of the following unfavorable prognostic factors: age, tumor location, histology, worsening functional status, and the limited efficacy of chemotherapy or radiation for malignant BSG.

Unfortunately, like other central nervous system gliomas, adult BSGs tend to recur after upfront RT and chemotherapy treatments with a latency that depends on histology [2]. Most patients have a rapid clinical response to initial therapy, and approximately 70% of patients experience improvement in neurological symptoms. However, overall treatment outcomes are disappointing, with nearly all patients manifesting disease within 5–8 months of RT [21]. Once the diagnosis of recurrent disease is established, salvage treatment is primarily based on medical therapy (in this case, the choice of pharmacological approach is also based on literature limited to a few studies conducted on a restricted number of patients). The response to salvage chemotherapy is also unsatisfactory, with a median OS of less than one year. Patients with progressive disease who receive salvage chemotherapy often have severe neurologic deficits and morbidity and have limited treatment options [21].

Disease control in BSG recurrences lasts only a few months and is unsatisfactory. Despite widespread use in the upfront setting and the good responses obtained, there are currently few cases in the literature on the use of re-irradiation in BSG recurrences in adult patients. Based on these grounds, the purpose of this systematic review is to evaluate re-irradiation as a potential choice in patients with relapsed adult BSG. Therefore, we will collect reported cases in the literature on the use of re-irradiation in BSG recurrences.

## 2. Materials and Methods

To identify relevant articles, we conducted searches on the PubMed and Scopus databases using the following terms: “adult—brainstem—gliomas—re-irradiation”. Articles retrieved were reviewed by GG and NG, and any discrepancies were discussed with FP. Only articles reporting clinical study results involving adult patients were included, while reviews, meta-analyses, case reports, and studies focused on pediatric patients were excluded. We also examined the bibliographies of identified papers from both databases to ensure comprehensive coverage.

For each included study, we assessed key parameters such as the number of patients analyzed, the dosage of ionizing radiation administered following radiological diagnosis or surgery, time to recurrence, re-irradiation dosage, survival post-second RT, and incidence of toxicity, when available.

## 3. Results

**Figure 1:**
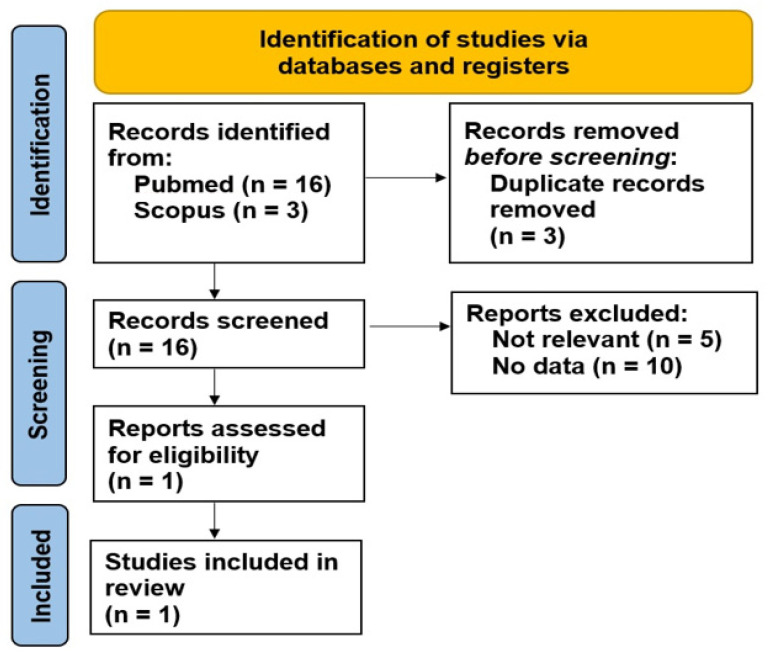
PRISMA flow diagram of this review.

Using the PubMed search engine, 16 studies were found. After reading the abstracts, only two of them were evaluable. Using the Scopus search engine, three studies appeared; however, only one paper (already selected using PubMed) could be considered for this analysis (Figure 1).

The first paper identified through our research was published by Amsbaugh et al. They reported in 2018 the results of a phase 1/2 study in patients with recurrent BSG [27]. The authors investigated the impact of three different radiation fractionations in 12 patients (24 Gy in 12 fractions, 26.4 Gy in 12 fractions, and 30.8 Gy in 14 fractions). Only one patient experienced grade 3 acute toxicity. Five out of six patients treated with 24 Gy in 12 fractions and one out of three treated with 30.8 Gy showed clinical improvement. The median survival from diagnosis was 30.8 months, and the PFS after re-irradiation was 4.5 months. The authors’ conclusion suggests that retreatment in recurrent brainstem glioma (BSG) cases is deemed safe and warrants consideration for patients facing this condition. Furthermore, their utility analysis indicates that a treatment regimen involving 24 Gy administered in 12 fractions may be preferable.

The second paper selected was published by Susheela et al. In 2013, they published the findings of a retrospective monocentric study carried out on five patients with recurrent BSG receiving re-irradiation [28]. All evaluated patients had poor performance status and neurological symptoms, and this bias may represent a potential critique of this article. Radiation doses ranged from 50 Gy in 25 fractions (three patients), 25 Gy in 5 fractions, and 50.40 Gy in 28 fractions after diagnosis. The time between the first and second radiotherapies varied from 12 to 26 months. After the second RT, patient survival was 3, 5, 6, 14, and 36 months, respectively. The patient who survived for 36 months developed toxicities related to retreatment, compromising their quality of life.

## 4. Discussion

Adult brainstem gliomas account for less than 2% of all gliomas. The standard therapeutic approach involves surgical intervention when feasible, often for biopsy purposes, followed by RT, depending on tumor histology [10,18]. Despite several efforts to design clinical studies, the rarity of this disease and the uncertainty associated with histological diagnosis make it challenging to investigate chemotherapy regimens for BSG. The infrequency of malignant tumors in adult patients makes it difficult to pinpoint prognostic markers and treatment response predictors. For instance, Guillamo et al. [2] conducted a translational study in 2001 to look into predictive markers for 48 adult patients with brainstem gliomas. Upon independent examination, the multivariate analysis revealed that variables such as the length of symptoms, the presence of “necrosis” on MRI images, and the tumor’s histological grade were significant predictors of prognosis (*p* < 0.05) (Table 1).

To identify the most effective treatment in child patients, Hargrave et al. [30] published a comprehensive review of 29 pontine clinical trials, showing disappointing survival results, with no systemic therapy showing benefit over conventional RT. Later, a prospective study was published on a pre-irradiation chemotherapy regimen based on hematotoxic and non-hematotoxic regimens [31]. This study provides impressive results for the operating system. Unfortunately, the survival benefit achieved with tamoxifen, BCNU, cisplatin, and high-dose methotrexate was due to a doubling of the length of hospital stay, significantly affecting patients’ quality of life [31]. Similarly, based on the promising results of the Stupp protocol [32], the Children’s Oncology Group (ACNS0126) decided to investigate the use of temozolomide simultaneously with RT followed by adjuvant temozolomide in patients with diffuse pediatric glioma in the treatment of glioblastoma; 1-year event-free survival was higher in the historical study used for comparison (albeit with a non-statistically significant *p* value) [33]. The Pediatric Brain Tumor Consortium investigated the use of capecitabine in combination with radiotherapy in children with diffuse pediatric glioma, reporting no improvement in PFS and OS [34].

Current North American clinical trials for pediatric patients with BSG use the dose of 59.4 Gy in 33 fractions (regardless of the volume of the pons involved). For smaller volumes of the brainstem (1–10 mL), irradiation to maximum doses of 59 Gy (2 Gy/fraction) may be feasible; however, the risk of neurotoxicity appears to significantly increase at doses exceeding 64 Gy [35]. Although radiation-induced brainstem injury is rare, its severity has garnered increased attention. Affected patients may present with cranial nerve impairment and symptoms indicative of long-tract (spinothalamic and corticospinal) and cerebellar injuries. While mild cases may be asymptomatic, more severe manifestations can include limb weakness, hemiplegia, gait instability, temperature sensory disturbances, diplopia, dysarthria, and facial and tongue paralysis [36]. Hyperfractionated radiotherapy has not demonstrated any benefit over standard fractionation; Farmer et al., in their review published in 2001 [11], found that hyperfractionated radiation therapy for diffuse pontine gliomas showed no benefit over a ten-year study period.

Ineluctably, adult brain stem gliomas recur after a period that varies depending on the histology of the neoplastic lesion, available therapies become less effective, and patients have a poor prognosis. RT causes the tumor to develop necrotic and cystic alterations, which could be mistakenly linked to a progressive disease. Differentiating post-treatment changes from anaplastic transformation and recurrent and progressive disease can be challenging, although advanced MRI sequences may help to reach the correct diagnosis [36,37,38,39,40,41]. Re-irradiation of the brainstem after initial RT of 50–55 Gy may be associated with significant, potentially fatal toxicity and should be approached with caution [26]. Few data have been published on the effects of brainstem re-irradiation on the development of necrosis, but some data from re-irradiation studies can be extrapolated to other brain regions [37]. Merchant et al. [37] reported that several pediatric patients with recurrent ependymoma were treated with re-irradiation and necrosis was observed, especially after stereotactic radiation. The incidence of necrosis appeared to be higher after using hypofractionated RT [25,26,27,28]. Nieder et al. [38] reported an incidence of confirmed radionecrosis of 2 among 16 adult patients treated with cumulative doses of 86 Gy or more on the central nervous system. Typically, acutely reactive tissues recover from radiation damage within a few months and tolerate a second entire course of radiation [39]. The risk of late toxicity increases with a higher cumulative dose, a larger volume of re-irradiated tissue, and a short treatment interval [40]. The first-line treatment for the occurrence of necrosis secondary to radiotherapy is steroid therapy, which, unfortunately, has significant side effects. Bevacizumab has shown a role in controlling radionecrosis in glioma patients. Despite its limited use in treating radionecrosis following radiotherapy in patients with BSG, bevacizumab could potentially contribute to managing radionecrosis in this setting. Liu et al., for instance, in 2009, reported a limited experience with four pediatric patients with BSG who developed radionecrosis after radiotherapy Three of the four patients benefited and were able to discontinue steroid therapy (one patient did not benefit due to disease progression).

Mayer and Sminia [40] reported that radiation-induced necrosis of normal brain tissue was observed at normalized total doses (NTDs (cumulative) > 100 Gy). When the irradiation technique is changed from conventional to radiosurgery retreatment, the applied re-irradiation dosage and NTD (cumulative) rise, but the likelihood of typical brain necrosis does not increase. Current treatment options allow re-irradiation of the brain in the palliative treatment of recurrent high-grade glioma with a reasonable probability of exposure to a limited amount of normal brain tissue radionecrosis.

Following the diagnosis of disease recurrence, therapeutic options are limited since salvage surgery is hardly feasible, second-line therapies are poorly effective, and targeted therapies need improvement [42,43]. RT is crucial in disease management post-diagnosis and is vital for disease control. In cases where central nervous system tumors are located outside the brainstem, re-irradiation becomes a consideration as a salvage option upon disease recurrence [44,45]. Although evidence from randomized studies is lacking and reported outcomes may be influenced by patient selection bias, the results regarding safety and disease control are promising, especially given the poor prognosis of patients with recurrent high-grade gliomas [42,46,47,48,49,50,51,52].

Despite the limited literature, re-irradiation is gradually emerging as a potential strategy for managing recurrent or progressive brainstem gliomas. In a recent systematic review, the benefit of re-irradiation was reviewed from seven studies, and it was found that, in pediatric diffuse intrinsic glioma, it may be considered by considering the cost–benefit balance A single-institution retrospective analysis of five adults with progressive/recurrent brainstem glioma treated with a repeat course of radiation resulted in post-treatment survival ranging from 3 to 36+ months. Four of the five patients showed improvement in performance status post-treatment, with the other patient manifesting new symptoms potentially attributable to radiation toxicity [28].

For patients with diffuse intrinsic pontine glioma, germinoma, medulloblastoma, and recurrent ependymoma, re-irradiation is a crucial component of salvage therapy. Conventionally fractionated re-irradiation (1.8 Gy/day) can effectively control the disease over the long term with minimal high-grade damage in patients with ependymoma [48]. Re-irradiation effectively relieves symptoms and increases survival for children with progressive diffuse intrinsic pontine glioma as compared to those who do not receive re-irradiation treatment. On the other hand, for individuals with medulloblastoma, repeat radiation therapy with craniospinal irradiation, if safe to administer, may offer long-term tumor control.

In light of these considerations, this investigation aimed to investigate published experiences with re-irradiation in adult patients with recurrent brainstem gliomas. The systematic review portion of this study narrowed down the selected experiences to two studies, comprising a total of 17 patients (12 treated in a phase 1 and 2 study and five studies in a retrospective experience). The authors of these studies reported a survival gain for treated patients of several months with acceptable toxicity. Notably, only one patient, who survived for 36 months, experienced significant toxicity due to retreatment.

While the data reported by the studies in this review are encouraging regarding the potential benefits of re-irradiation in patients with recurrent brainstem gliomas, the limited number of patients included makes it challenging to draw definitive conclusions regarding safety. Compared to adult patients, there is a more significant body of evidence regarding re-irradiation in pediatric patients with recurrent brainstem glioma (BSG). For example, Lassaletta et al. [49] conducted a retrospective multicenter study in 2018 involving 16 pediatric patients. The authors reported a median survival of 6.4 months from the completion of the second RT and noted good tolerability of re-irradiation. In this study, a dosage ranging from 21.6 to 36 Gy was administered during the second irradiation. Several cases demonstrated clinical improvements, and the treatment was well tolerated [49]. Additionally, Krishnatry et al. [50] published, in 2020, the results of a retrospective study involving 20 pediatric patients who underwent re-irradiation following the diagnosis of recurrent BSG. The study found that 85% of treated patients experienced clinical benefit from retreatment without encountering toxicity greater than grade 2.

Immunotherapy is emerging as a promising new modality for treating these gliomas. Morimoto et al. [52] reported the first case of BSG treated with a combination of RT and autologous formalin-fixed tumor vaccine (AFTV) in a 32-year-old man presented with left facial numbness and right hemiparesis. MRI conducted at 42 months after the combination therapy showed a 91% decrease in tumor volume, and the regression was maintained for five years; the patient died 83 months after diagnosis. Similarly, nonspecific passive immunotherapy involves the administration of agents or activated effector cells to nonspecifically activate the immune system to produce anticancer effects [53]. For example, this processing can be carried out by cytokines or lymphokine-activated killer cells (LAK cells). Cytokines are low-molecular-weight proteins that play an essential role in all phases of the immune response, both humoral and cellular. To achieve a biological effect, the cytokine must bind to a specific receptor on the target cells (T and B lymphocytes, natural killer cells, monocytes/macrophages, and granulocytes) [53,54]. Antiangiogenic therapy may help these patients with BSG, as it also improves understanding of this rare disease and helps physicians seek more effective treatments [22]. Antiangiogenic treatment can restore the normal structure and function of tumor vasculature, which enhances drug delivery and returns the tumor microenvironment to normal [23]. Bevacizumab, an anti-vascular endothelial growth factor monoclonal antibody that has been thoroughly researched in recurrent glioblastoma, is one example of an antiangiogenic medication that has been reported to be an effective salvage treatment for progressive BSG in recent years [24]. Bevacizumab administration has been associated with improved clinical outcomes, acceptable radiologic responses, and a PFS of up to two years [25]. However, for adult BSG, the effectiveness of first-line antiangiogenic treatment is uncertain [22].

The fact that multiple targeted therapies for BSGs failed during their late clinical development shows that most of these cases are not even close to being driven by a single pathway, making them candidates for targeted therapy [55]. It is important to consider the role of genetic mutation variants, which can provide more specific targets when designing personalized treatment in select patients [15,16,17,18,19,20,21,22,23,24,25,26,27,28,29,30,31,32,33,34,35,36,37,38,39,40,41,42,43,44,45,46,47,48,49,50,51,52,53,54,55,56]. To make meaningful go/no-go judgments for additional clinical research, better clinical trial design and early incorporation of control arms in phase II settings are required. Moreover, platform trials investigating several compounds might theoretically hasten the development of new drugs. It is significant to remember that targeted therapies for BSG are still in the early phases of research and development, and it is unknown how safe and effective they will be in the long run. In this situation, obstacles like tumor heterogeneity, resistance mechanisms, and blood–brain barrier penetration could make targeted therapies less effective. However, the discovery of molecular targets and genetic modifications has created new opportunities for precision and personalized medicine strategies, and ongoing studies and clinical trials indicate that better treatment options will be available in the future. Finally, liquid biopsy exhibits potential as a diagnostic and monitoring tool for BSGs; however, its applicability is limited by factors such as tumor type, location, and molecular characteristics, which can affect liquid biopsy’s sensitivity [51]. Additionally, since ctDNA can be obtained from non-tumor tissues or non-malignant cells, identifying actionable targets from liquid biopsy necessitates careful interpretation and validation [51].

## 5. Conclusions

Adult BSG is a dismal condition, and consequently, any intervention that may improve the quality of life, clinical course, and survival outcomes should be given greater attention as a feasible option. Pooled data from the available investigations support the idea that re-irradiation is one of these alternatives. In the absence of therapeutic alternatives capable of impacting the progression of brainstem gliomas, re-irradiation could be considered for prospective studies designed to assess its safety and benefits. The dose of 24 Gy in 12 fractions appears to be the most promising for further research. Considering the prognosis of these patients and the limited benefit in terms of months from the published studies, it is important to involve patients and their caregivers in the therapeutic decision-making process. It should also be specified in the informed consent that potential survival benefits may be associated with significant treatment-related toxicities.

## Figures and Tables

**Table 1:** Summary of most frequent types of brainstem gliomas in adults (data from Guillamo et al.) [2].

	Low Grade Diffuse Intrinsic Gliomas	Malignant Intrinsic Gliomas
Frequency	46%	31%
Age of onset	20–30 years	>40 years
Duration of symptoms	>3 months	<3 months
Clinical presentation	Facial palsy, diplopia, ataxia	Dependent on location
Location	Pons, medulla	Variable
MRI features	Diffuse, without contrast enhancement	Enhancing mass with central necrosis
Histology	Low grade (II)	High grade (III–IV)
Treatment	RT	RT
Median survival	Seven years	One year

RT, radiotherapy.

## Data Availability

The authors confirm that the data supporting the findings of this study are available within the article.
